# Single-cell screening of photosynthetic growth and lactate production by cyanobacteria

**DOI:** 10.1186/s13068-015-0380-2

**Published:** 2015-11-25

**Authors:** Petter Hammar, S. Andreas Angermayr, Staffan L. Sjostrom, Josefin van der Meer, Klaas J. Hellingwerf, Elton P. Hudson, Haakan N. Joensson

**Affiliations:** Science for Life Laboratory, Division of Proteomics and Nanobiotechnology, KTH Royal Institute of Technology, Stockholm, Sweden; Novo Nordisk Foundation Center for Biosustainability, KTH Royal Institute of Technology, Stockholm, Sweden; Swammerdam Institute for Life Sciences, University of Amsterdam, Amsterdam, The Netherlands; Institute of Science and Technology (IST) Austria, Klosterneuburg, Austria

**Keywords:** Cyanobacteria, Single cell, Droplet microfluidics, High-throughput screening, Lactate dehydrogenase

## Abstract

**Background:**

Photosynthetic cyanobacteria are attractive for a range of biotechnological applications including biofuel production. However, due to slow growth, screening of mutant libraries using microtiter plates is not feasible.

**Results:**

We present a method for high-throughput, single-cell analysis and sorting of genetically engineered l-lactate-producing strains of *Synechocystis* sp. PCC6803. A microfluidic device is used to encapsulate single cells in picoliter droplets, assay the droplets for l-lactate production, and sort strains with high productivity. We demonstrate the separation of low- and high-producing reference strains, as well as enrichment of a more productive l-lactate-synthesizing population after UV-induced mutagenesis. The droplet platform also revealed population heterogeneity in photosynthetic growth and lactate production, as well as the presence of metabolically stalled cells.

**Conclusions:**

The workflow will facilitate metabolic engineering and directed evolution studies and will be useful in studies of cyanobacteria biochemistry and physiology.

**Electronic supplementary material:**

The online version of this article (doi:10.1186/s13068-015-0380-2) contains supplementary material, which is available to authorized users.

## Background

Cyanobacteria are model organisms for the study of biological light harvesting [1], photosynthesis [2], and circadian gene regulation [3]. Additionally, their minimal nutrient requirements and an expanding genetic toolbox have made cyanobacteria attractive for a range of biotechnological applications, such as hosts for next-generation biofuel [4, 5] and fine chemical production [6].

Microfluidic droplet emulsion technology [7, 8] is emerging as a powerful facilitator for cellular and metabolic engineering, as various devices enable precise single-cell compartmentalization [9], culturing [10], phenotyping [11], and sorting [12]. Recent works have demonstrated the assay and enrichment of microbial and mammalian clones based on substrate consumption [11], secreted proteins [13, 14], and metabolites [11]. Additionally, single-cell technology allows study of population heterogeneities in metabolism and can thus potentially uncover rare metabolic states not visible in bulk assays [15].

Here we report a droplet microfluidic workflow which combines several microfluidic devices to encapsulate, assay, and sort l-lactate-producing strains of the cyanobacterium *Synechocystis* sp. PCC6803 *(Synechocystis).* A UV-mutagenized population was sorted and a clear enrichment of high-producing strains was observed. We also used the droplet platform to measure population heterogeneities in *Synechocystis* growth and l-lactate production and their dependence on a circadian dark–light cycle.

## Results and discussion

**Fig. 1:**
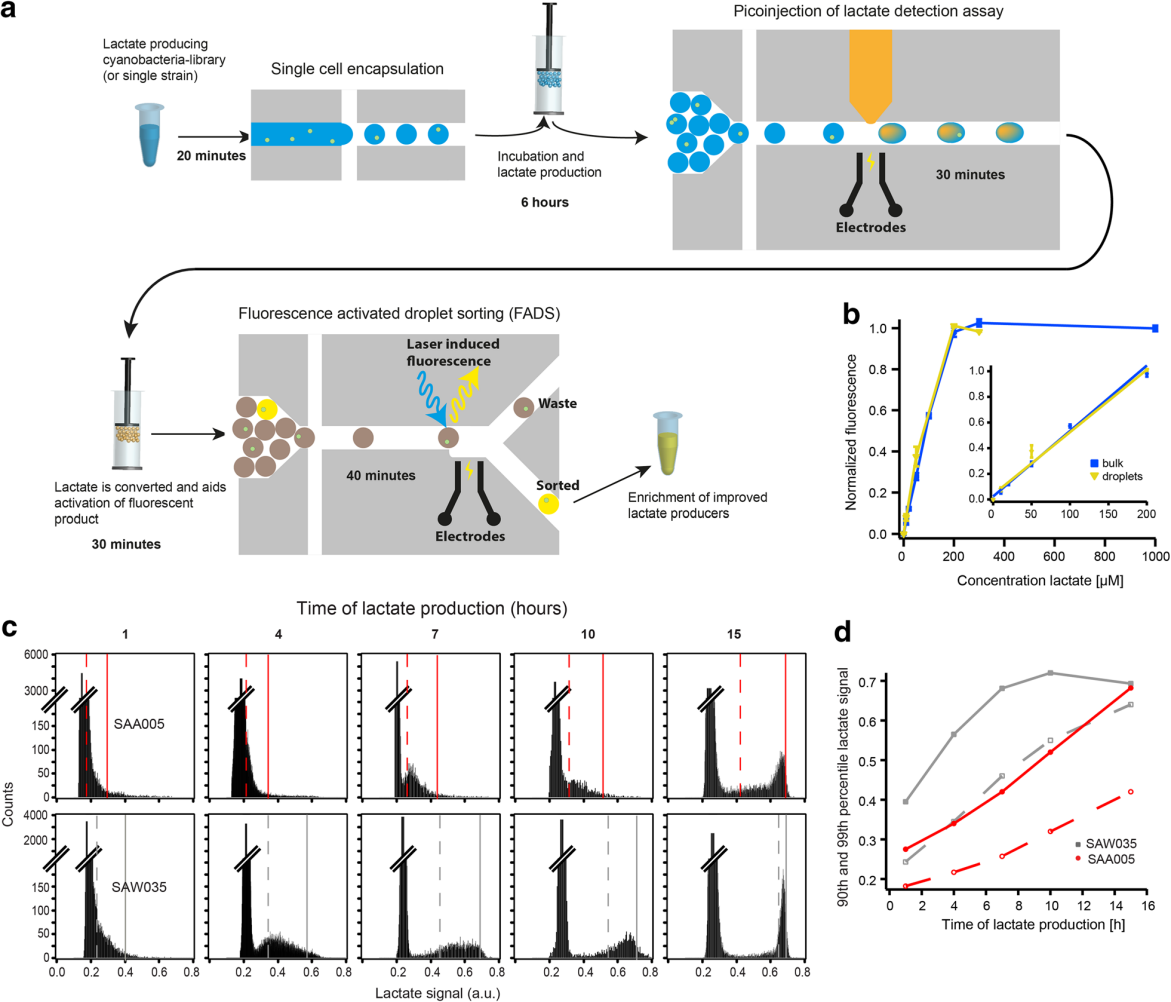
Experimental workflow. **a** Lactate-producing cyanobacteria are encapsulated as single cells in 10 pL droplets. Following incubation to produce lactate, the droplets are injected with an enzyme assay that catalyzes the activation of a fluorescent dye in the presence of lactate. The fluorescent droplets are analyzed and sorted based on the strength of the signal. **b** Titration of pure lactate analyzed according to the workflow in ***a*** (*yellow triangles*) and using microtiter plate (*blue squares*). **c** Time-lapse experiment of lactate-producing strains (*n* = 10,000 droplets analyzed per time point, *y*-axis is broken). *Vertical continuous* and *dashed lines* mark the 99th and 90th percentiles, respectively. **d** Analysis of data in **c**, showing the 99th percentile (*continuous lines*, *filled markers*) and 90th percentile (*dashed lines*, *open markers*) for SAW035 (*gray squares*) and SAA005 (*red circles*)

The workflow begins with encapsulation of single *Synechocystis* cells in picoliter droplets (10 pL) containing growth medium (Fig. 1a). The resulting droplet emulsion is incubated under light, where l-lactate (lactate) is produced and secreted within each droplet. Droplets are loaded onto a second device, where a picoinjector [16] adds a lactate assay enzyme solution (10 pL) into each droplet via electrocoalescence, resulting in a fluorescence response. Picoinjection is preferable to co-encapsulation of cells with assay solution because it decouples photoautotrophic lactate production (6 h) from the lactate assay (30 min), thus allowing the use of assay reagents that may be transported out of droplets on a longer timescale or that are light sensitive. After picoinjection, droplets are screened (10^3^ droplets/s) and sorted (10^2^–10^3^ droplets/s) in a third device using fluorescence-activated droplet sorting (FADS) [17]. The sorted emulsion is spread on agar plates for colony formation and counting after incubation in light. Calibration assays using pure lactate (no cells) showed a linear response from 10 to 200 μM lactate, which is comparable to a 50 μL microtiter plate assay using the same reagents (Fig. 1b; Additional file 1: Fig. S1). Fluorescence distributions of calibration samples (no cells) provided a measure of total technical variation of the encapsulation, picoinjection, and enzymatic assay steps (Additional file 1: Fig. S2a–c). Details are available in the “Methods” section and the additional file 1.

We sought to determine the incubation time that maximizes assay resolution between lactate-producing *Synechocystis* strains. Two engineered *Synechocystis* strains, SAA005 and SAW035, were selected as low and high lactate producers, respectively [18, 19]. In microtiter plates, SAW035 has a 5.5 ± 1.9-fold (mean ± SEM, *n* = 3) higher productivity than SAA005 (Additional file 1: Fig. S3a). We incubated SAA005 and SAW035 in droplets and assayed them for lactate production at different time points. For SAW035, the droplet lactate content was within the linear dynamic range of the assay at 1–10 h of incubation (4–15 h for SAA005) (Fig. 1c). The differences were shown at the 99th and 90th percentiles for all time points (Fig. 1d). The best resolution was at 4–7 h incubation time, where the lactate titer for SAW035 was 4.3 ± 1.4-fold (mean ± SEM, *n* = 3) higher than for SAA005 (Additional file 1: Fig. S3a), similar to values obtained in a microtiter plate at the same experimental conditions. A subpopulation of cells from each strain did not produce lactate (see, e.g., Fig 1c). This fraction was consistently higher for SAW035 (27.3 ± 1.9 %) than for SAA005 (15.9 ± 2.8 %) (mean ± SEM, *n* = 3 and *n* = 5, respectively) (Additional file 1: Data and Notes).

**Fig. 2:**
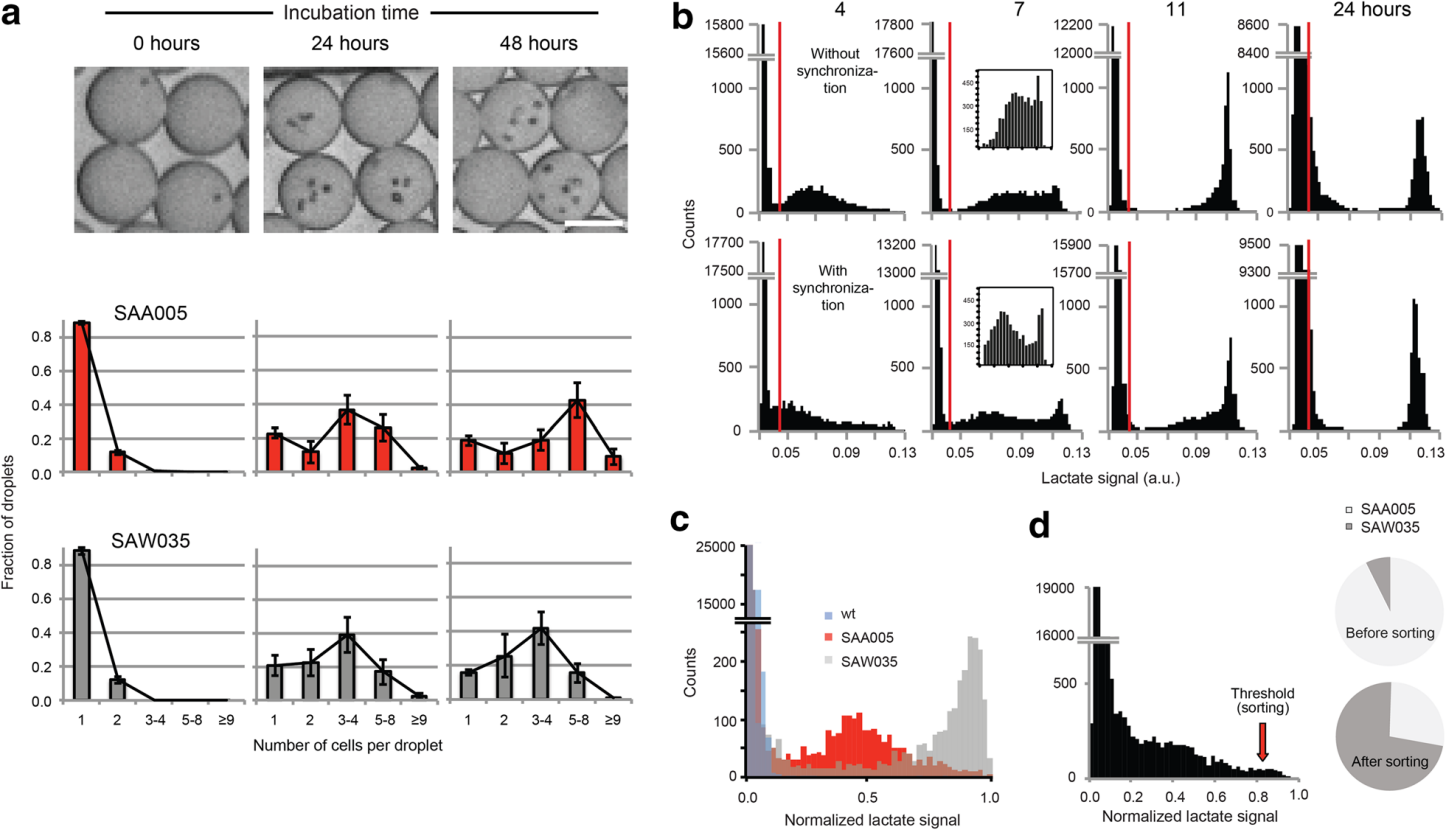
Viability in droplets and sorting. **a** Growth of encapsulated *Synechocystis* cells represented by photomicrographs, *scale bar* 20 μm (*top*), and counted cells per droplet (*graphs* show mean ± SEM) for strains SAA005 (*middle*; *n* = 5 independent experiments) and SAW035 (*bottom*; *n* = 3). **b** Lactate production in strain SAA005 with and without a 12-h pre-incubation in darkness. *Red* *vertical lines* are added as visual reference (*n* = 30,000 droplets analyzed per time point, *y*-axis is broken). Insets at 7 h show re-binning of data in the *x*-axis interval 0.05–0.13. This is to visualize the difference between synchronization and no synchronization. **c** Histogram of lactate production for a mix of wild type, SAA005, and SAW035. **d** 12:1 model library of SAA005 and SAW035 shown as fluorescence from lactate (*left*) and enrichment in sorting (*right*)

The droplet platform also allows comparison of growth rates between different clones or strains [20, 21]. To estimate doubling times, we followed cell proliferation from single cells within droplets for both SAA005 and SAW035 (Fig. 2a). The average doubling time of SAA005 in the droplets was approximately 12 h, comparable to batch culture at similar pCO_2_ and light intensities [18]. The doubling time of SAW035 was longer, as expected. Under optimal conditions, this strain diverts up to 18 % of fixed CO_2_ to lactate (<1 % for SAA005) [19]. For both strains, we observed a subpopulation (10–30 %) that did not divide. We hypothesized that this subpopulation is the non-producing subpopulation observed in lactate assays. We confirmed this by simultaneously monitoring cell division and lactate production in droplets (Additional file 1: Fig. S4a–c). Non-dividing cells did not produce lactate, though a majority of cells were viable at the time of droplet encapsulation as determined by oxonol staining and during droplet incubation as determined by cellular autofluorescence (Additional file 1: Fig. S4d, e). These cells are therefore metabolically stalled on the time scale of this experiment (48 h).

The observed widths of the distributions of lactate concentration of *Synechocystis* are influenced by technical but mostly biological variability (Additional file 1: Fig. S2c–e) and were similar for SAA005 and SAW035. Biological heterogeneity could arise from cells being in different growth phases at the time of encapsulation. Different cells will also be in different states of the cell cycle, and a cell that is close to dividing will have almost double the size and more enzymes present compared to a cell that has just divided. High chromosomal copy number [22, 23], growth-phase-dependent DNA replication, and the apparently random chromosome partitioning at division [24, 25] can further add to the heterogeneity of *Synechocystis* gene expression. However, reduced heterogeneity would increase assay resolution. We attempted to reduce heterogeneity in lactate production of SAA005 by synchronizing gene expression and metabolism with circadian light–dark entrainment [26]. A 12-h dark period was introduced just prior to encapsulation and lactate production. We observed that cells subjected to darkness had a delayed onset of both lactate production and division, but did not exhibit narrower distributions of lactate production compared to those that were treated with continuous light (Fig. 2b; Additional file 1: Fig. S5, Data and Notes).

We next used the droplet platform to assay and sort mixtures of the lactate-producing strains. To estimate enrichment potential, a 1:1:1 mixture of wild-type *Synechocystis*, SAA005, and SAW035 was assayed. Each strain was barcoded with a known concentration of the dye fluorescein to allow identification (Fig. 2c). After incubation, wild-type droplets did not show detectable lactate levels, indicating no or negligible production. This also suggests that there is no substantial leakage of lactate in between droplets on this time scale. The barcode revealed overlap of SAA005 and SAW035 lactate distributions; a 0.5 % gating threshold contained 95 % of the high-producing SAW035 and 5 % SAA005 (Additional file1: Fig. S3b–d). Perfectly efficient sorting using the 0.5 % gate would thus be expected to enrich the SAW035 strain 38-fold (Additional file 1: Data and Notes). We created a 1:12 mixture of SAW035 and SAA005 and then sorted 5000 cells using the 0.5 % gating threshold and plated them (details in the “Methods” section; Additional file 1: Table S1). SAW035 was enriched 32-fold as quantified by colony counting (Fig. 2d). This enrichment is comparable to the expected outcome for these two strains, as estimated from the barcode experiment. The theoretically maximal enrichment for non-overlapping populations is 77-fold [17] (Additional file 1: Data and Notes). Three colonies were picked from the plate, and cultures derived thereof were confirmed to produce lactate as expected for the respective strains (Additional file 1: Data and Notes). Total assay time was approximately 8 h; total reagents per single cell were 10 pL each of cell medium and enzyme assay, i.e., 10^7^-fold lower than for a corresponding single clone in a 100 μL reaction [27].

**Fig. 3:**
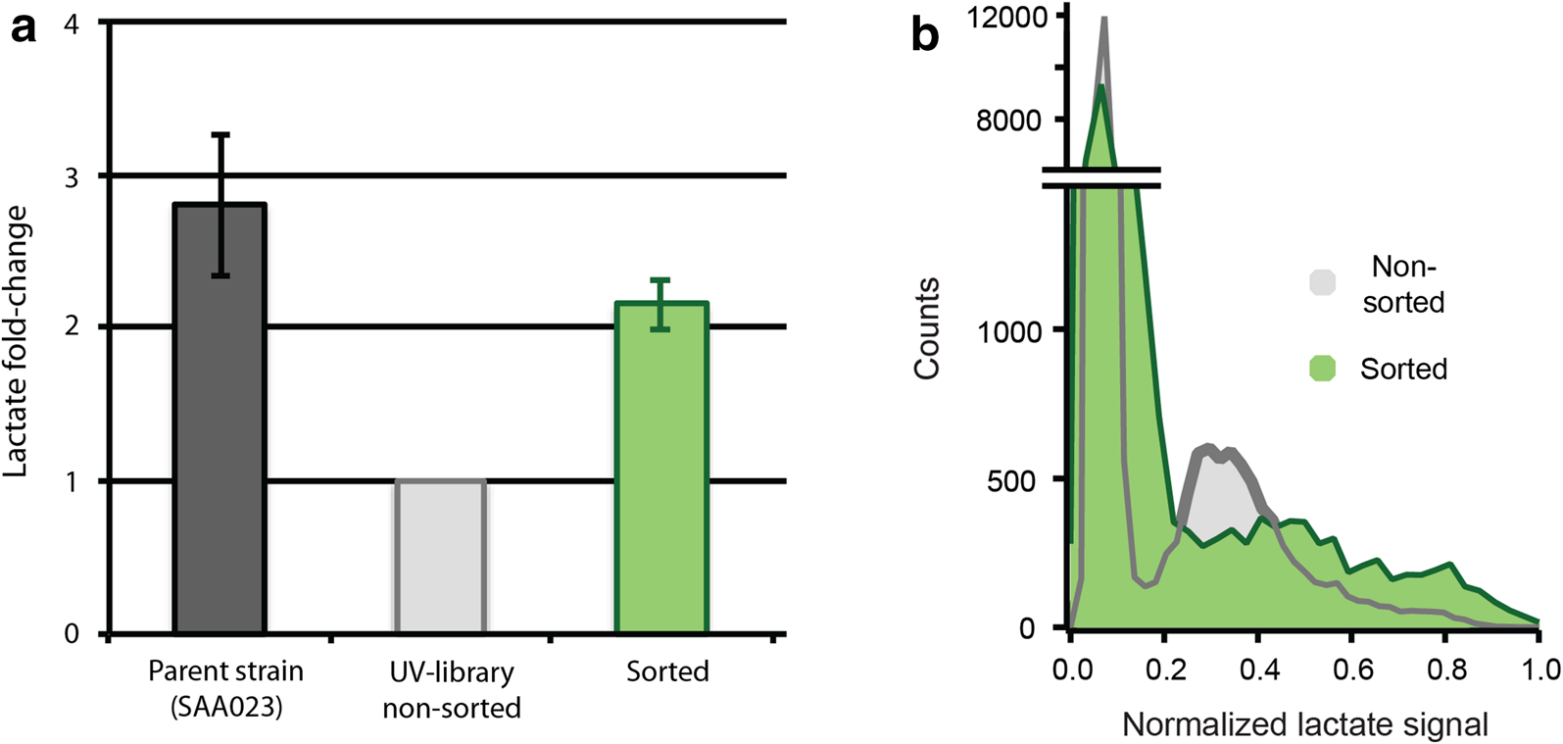
Sorting a large UV mutagenesis library. **a** Bulk lactate production measured in microtiter plate. Parent strain SAA023 is compared to the non-sorted UV-generated mixture and the droplet-sorted fraction (*n* = 5). Data represents technical replicates (the same sorted fraction screened for lactate at different days after sorting). Lactate production is normalized to that of the non-sorted population in each experiment and is shown as average ± SEM (*n* = 5). **b** Lactate production in droplets of the non-sorted (*gray*) and the sorted fraction (*green*); *n* = 30,000 droplets analyzed per sample, *y*-axis is broken. A clear enrichment of high-producing strains is observed

Finally, a UV-mutagenized population (estimated 4 × 10^5^ members) was created by UV irradiation of the lactate-producing strain SAA023 [18]. The UV irradiation was such that 90–99 % of cells were killed. The UV-irradiated population had a lower average lactate production than the parent strain SAA023 (Fig. 3a). This indicates that UV exposure triggers many harmful mutations that affect growth and lactate production of the cells. The mixture was screened and sorted using a similar procedure as above. A 3 % gating threshold was used, and the sorted fraction was recovered on an agar plate which resulted in >10,000 colonies (out of estimated 20–40,000 sorted). An aliquot of the non-sorted library emulsion was plated in the same way and used as a reference sample. The sorting significantly enriched high lactate producers of the UV-mutagenized population (Fig. 3a). The difference in lactate production for the non-sorted and sorted population was also seen when screened in droplets (Fig. 3b).

## Conclusions

We have developed an assay for single-cell screening of lactate production from the cyanobacterium *Synechocystis*, where droplet encapsulation allows us to couple genotype to production phenotype. We envision the droplet microfluidics platform as a powerful tool with many applications in applied and basic research. Enzymatic, fluorescence-based assays for a range of secreted metabolites have been devised [11]. Simultaneous measurement of productivity and growth adds a new dimension to strain selection from libraries. Furthermore, droplet microfluidics modules are amenable to automation, such that all steps of a directed evolution program could be integrated on a single device and automated; we observed clonal expansion in droplets over several days and emulsion breaking did not harm cells. Such a workflow would eliminate time-consuming plating and colony formation. In particular, for cyanobacteria, droplets can be arranged in monolayers or mixed, ensuring uniform light intensity during incubations.

## Methods

### Strain information and growth conditions

The cyanobacterial wild-type strain used in this study is the glucose-tolerant derivative of *Synechocystis* sp. PCC6803 (kindly provided by Devaki Bhaya, Stanford, USA). Heterologous insertions were made for the construction of the three lactate-producing strains employed here. Briefly, for the low-producing strain, SAA005, the native L-*ldh* gene of *Lactococcus lactis* ssp. *cremoris* (*L. lactis*) and a kanamycin resistance cassette were inserted downstream of the *psbA2* open reading frame in the *Synechocystis* genome [18]. For the medium-producing strain, SAA023, a codon-optimized version of the *L. lactis ldh* gene was flanked by the promoter P*trc* and a transcriptional terminator fused to a kanamycin resistance cassette and inserted into the genome of *Synechocystis* targeting *slr0168* [18]. For the high-producing strain, SAW035, the codon-optimized *ldh* gene of *L. lactis* was fused to P*trc* and cloned into the pDF-plasmid, and transformed into *Synechocystis* by bacterial conjugation as reported in [19]. Cells were cultivated in BG-11 supplemented with 50 mM NaHCO_3_ (pH 8) (BG-11-NaHCO_3_) and 20 µg/ml kanamycin (SAA005 and SAA023) or 25 μg/ml spectinomycin (SAW035) in flasks shaking at 180 rpm at 30 °C (CLF Plant Climatics) and illuminated with white light (30–35 μE/m^2^/s). Solid medium plates were prepared by adding 1.5 % (w/v) agar, 10 mM TES-KOH (pH 8) and 0.3 % (w/v) sodium thiosulfate to BG-11, and antibiotics were added if appropriate. During growth of the SAA023-derived UV-mutagenized cells, kanamycin was always present and no NaHCO_3_ was added.

### Microfluidic chip manufacturing

Figure 1a illustrates the overall experimental setup. Microfluidic chips were designed and manufactured in polydimethylsiloxane (PDMS) (Dow Corning) as described in [14] for the sorting chip, and in [28] for the generation and picoinjector chips with the following modifications: the droplet generation chip had a nozzle width of 25 μm; the picoinjector chip had reinjection and picoinjection nozzle widths of 22 and 16 μm, respectively, and a channel depth of 25 μm. The devices were bonded on glass slides using oxygen plasma, the electrodes were fabricated (when applicable) by injecting low-melting point solder (Indium Corp.), and the channels of the chip were surface treated with Aquapel (PPG Industries) followed by pressurized air.

### Droplet generation

*Synechocystis* cultures were started from plate and incubated for 3–4 days in batch. 12–24 h before harvesting, the cultures were diluted to assure growing cells with an OD_730_ of 0.4–0.6 at the time of harvest. Upon harvesting, the cells were washed twice in BG-11 medium and resuspended in BG-11-NaHCO_3_ to OD_730_ = 0.2–0.3. Except for in the sorting experiments, prior to droplet encapsulation the separate strains (or pure lactate samples) were mixed with different concentrations of fluorescein (Sigma-Aldrich) (0 for wild type, 2 for SAA005, and 10 μM for SAW035) to allow discrimination according to the fluorescence signal (laser excitation at 491 nm, emission filter 525/20 nm) and kept in 1-mL plastic syringes (BD plastic). A mix of HFE-7500 oil and 1 % (w/w) EA surfactant droplet stabilizer (RainDance Technologies) was loaded into a Gastight 5-mL glass syringe (Hamilton). Cells were encapsulated in 10 pL droplets essentially as reported earlier [14] using a flow rate of 400 μL/h for the aqueous solution and 2000 μL/h for the oil. The emulsion was collected in a 1-mL plastic syringe at a withdrawal flow rate of 2000 μL/h. The syringes were connected to the chip by polyether ether ketone tubing, and flow rates were controlled by neMESYS syringe pumps (Cetoni GmbH). The initial cell concentration used gives on average 0.2 cells per droplet and about ten times more droplets with one compared to two cells at the time of droplet generation. 1 cell per 10 pL droplet corresponds to OD_730_ = 1 (Additional file 1: Data and Notes). The different strains or lactate samples were encapsulated sequentially and collected in the same syringe, or mixed prior to washing and encapsulated within 30 min (time between washing and last droplet encapsulated, resembling a library situation). The emulsions were kept in syringes, placed under light (30–35 μE/m^2^/s), and incubated for lactate production as indicated for each experiment. For the lactate titration experiment (Fig. 1b), no incubation was needed.

### Lactate detection assay

Following incubation, the picoinjector chip [28] was used to supplement the droplets with a l-lactate assay mixture (Cayman Chemical Company). In brief, 1× solutions of the kit reagents were prepared according to the manufacturer’s instruction and mixed as 1 volume cofactor mixture, 1 volume fluorescent substrate, 2 volumes of enzyme mixture, and 1 volume bovine serum albumin (BSA, 1 % w/v, not supplied in the kit). BSA was added to slow the leakage of the fluorescent product between droplets. The assay mixture was loaded into a 1-mL plastic syringe. When picoinjecting, the droplet emulsion was injected to the chip at a flow rate of 70 μL/h, the oil–surfactant mix separated the droplets (supplied at a flow rate of 500 μL/h), and the enzyme mix was added at a flow rate of 40 μL/h. The droplets and enzyme mix met at a channel junction next to the in-built electrode, and droplets and enzyme mix were fused at a 1:1 volume ratio to form 20 pL droplets when an electrical field was applied (constant applied voltage of 0.6 kV at 30 kHz). The processed droplets were collected in a 1-mL plastic syringe at a withdrawal flow rate of 550 μL/h, and incubated (in darkness) for 30–40 min after collection was finished. In the enzymatic reaction, lactate dehydrogenase catalyzes the oxidation of l-lactate into pyruvate, and NAD^+^ is reduced to NADH+H^+^. In the subsequent step, NADH reacts with and activates the fluorescent substrate.

### Detection and sorting

Droplets were injected into the sorting chip at a flow rate of 30 μL/h and were separated by the oil–surfactant mix supplied at a flow rate of 500 μL/h. The droplets passed a 491 nm laser beam (Cobolt Calypso CW <100 mW), which was focused through a 10× objective lens onto a point close to the built-in electrode. Fluorescence was detected using two separate emission filters (525/20 nm for fluorescein and 593/20 nm for the lactate fluorescent substrate) and photomultiplier tubes (PMTs) (Hamatsu). The main channel of the chip splits into two just downstream of the excitation/electrode point (Fig. 1a, bottom), one channel leading to a waste vial and one leading to a collection port connected to a 1-mL plastic syringe, set to a withdrawal flow rate of 165 μL/h. The PMTs were connected to a Field Programmable Gate Array (National Instruments) programmed to activate a voltage pulse when the fluorescent signal detected exceeded a gating intensity. The voltage pulse was then amplified in a high-voltage amplifier (TREK Inc.) connecting to the built-in electrodes. The amplified voltage pulse (0.8–1 kV, 800 μs, 30 kHz) routed the droplet to the collection syringe, while in the absence of a pulse droplets would follow the default route to the waste.

### Strain verification and enrichment quantification

After sorting, the collected emulsion was mixed with emulsion breaker (1H,1H,2H,2H-perfluoro-1-octanol, Sigma-Aldrich) and medium to separate the cells from the oil, and spread on BG-11 plates. Equal aliquots were spread on plates containing kanamycin or spectinomycin. The two strains strictly grow on their selective condition (SAA005 has only kanamycin resistance and SAW035 has only streptomycin/spectinomycin resistance). Following 10 days of incubation, colonies were counted and the enrichment quantified (Additional file 1: Data and Notes).

### Library construction and sorting

The mixture of UV-mutagenized SAA023 cells was generated as follows [29]. UV irradiation of the SAA023 strain was done in an Amersham Bioscience UVC 500 DNA cross-linker with 4 × 8 W UV-B bulbs (emission 254 nm). Three sub-libraries of different UV irradiation were created: 60, 75, and 100 J/m^2^. For each sub-library, 250 μL of SAA023 culture at OD_730_ = 2 was aliquoted into a 40-mm petri dish and exposed to the UV dosage. To prevent photolyase-mediated DNA repair, cells were kept in the dark until plated (250 μL) on BG-11 agar plates containing kanamycin. The plates were then covered with Asmetec SFG10 520 nm filter foil and placed in an incubator at 30 μE/m^2^/s white light, 30 °C, and 1 % CO_2_. An estimated 5 × 10^3^ (100 J/m^2^) and 5 × 10^4^ (60 and 75 J/m^2^) colonies per plate were recovered after 2 weeks of cultivation for each UV intensity. An untreated sample gave approximately 5 × 10^5^ colonies. Eight replicates of each sample were made, so that the total library size was approximately 4 × 10^4^ (100 J/m^2^) and 4 × 10^5^ (60 and 75 J/m^2^). The UV-treated cells were scraped from the plates, resuspended in BG-11 medium containing 7 % v/v DMSO, and stored at −80 °C.

The three fractions irradiated with increasing intensity (60, 75, and 100 J/m^2^) were started from glycerol stocks 1 week prior to the first sorting experiment. Mixing these at equal ratios created our UV library. This mixture was washed and encapsulated in droplets as described above. Picoinjection and droplet sorting was performed following 6-h incubation in light, screening 3 × 10^5^ cells and using a gating threshold of 3 % of all droplets. The sorted fraction was spread on BG-11 agar plates containing kanamycin. An aliquot of the remaining non-sorted emulsion was also plated and used as reference in all subsequent measurements. After 2 weeks of incubation, colonies were scraped from the plates and cultured in liquid medium for another week. Lactate production was measured in microtiter plate and in droplets.

### Growth in droplets

Cells of SAA005 and SAW035 were encapsulated in droplets and collected in separate syringes as described above. Droplets were imaged at generation, or after 24 and 48 h of incubation and reinjection into a new microfluidic device, using 10× optical magnification where essentially all cells are observed in the same focal plane, and a Stingray F-033B camera (Allied Vision). Analysis was performed through manual counting of cells in droplets. One limitation in the analysis is to separate nearby cells, and data are therefore presented with binning, assuming full cell cycle events (for example, ‘5–8 cells’ means a single cell has divided three times).

### Synchronization experiment

Growth in droplets and lactate detection was performed as described above; however, samples were covered in aluminium foil and incubated for 12 h prior to encapsulation in droplets. Analysis was done after 0, 12, 24, and 48 h of incubation.

## Additional file

10.1186/s13068-015-0380-2 Supplementary information.

## Abbreviations

FADS: fluorescence-activated droplet sorting
Lactate: l-lactate
*Synechocystis*: *Synechocystis* sp. PCC6803
PDMS: polydimethylsiloxane
PMT: photomultiplier tube
