# Supplementary material for: Single-cell screening of photosynthetic growth and lactate production by cyanobacteria
Source: Biotechnol Biofuels. 2015 Nov 25;8:193. doi: 10.1186/s13068-015-0380-2 (PMC4660834; doi:10.1186/s13068-015-0380-2)
Supplement: Supplementary file 1 — 10.1186/s13068-015-0380-2 Supplementary information. [file 13068_2015_380_MOESM1_ESM.pdf]

Additional file 1
Supplementary Information

Single-cell screening of photosynthetic growth and lactate production by cyanobacteria

Hammar *et al.*

Supplementary Figures 2
Supplementary Tables 7
Supplementary Data and Notes 8
1. Growth and viability in droplets 8
2. Lactate production in droplets and batch culture 8
3. Variability in droplet detection 9
4. Quantifying sorting efficiency 10
5. Supplementary Methods 10
Fluorescence microscopy 10
Live/dead staining and fluorescence microscopy 10
Fluorescence microscopy for lactate production 11
Optical density normalization 11
Microtiter plate measurements 11
6. Supplementary references only 11

Supplementary Figures

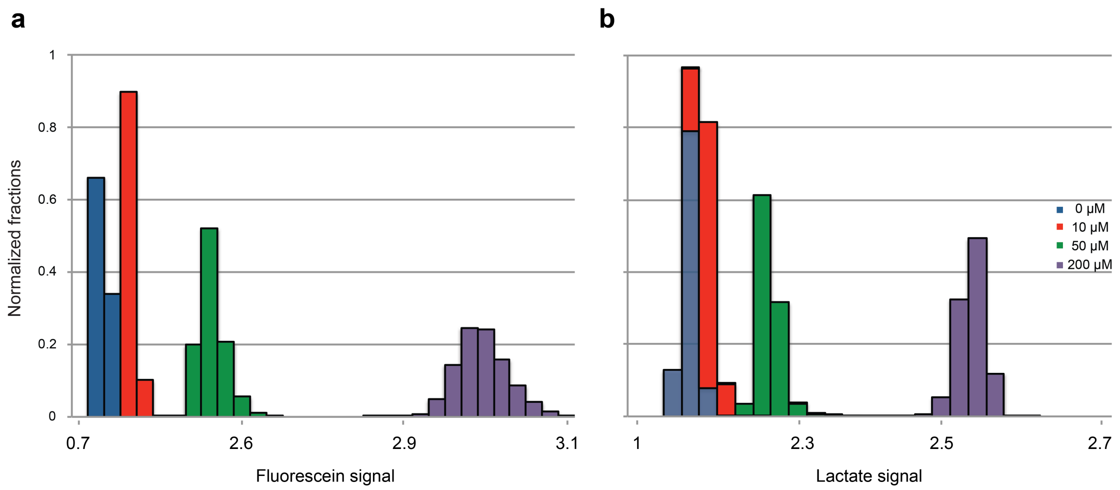

Fig. S1 Representative data set of analyzed droplets as shown in Figure 1b. Fluorescence from different concentrations of **(a)** fluorescein and **(b)** the substrate of the L-lactate assay, corresponding to 0 (blue, n=29,335 droplets), 10 (red, n=31,675), 50 (green, n=30,633) and 200 (purple, n=29,984) µM lactate. Respective distribution in *b* is obtained by gating in *a*, as seen from the color-coding. Data is binned and plotted in log-scale. Each subpopulation is normalized to the sum of 1.

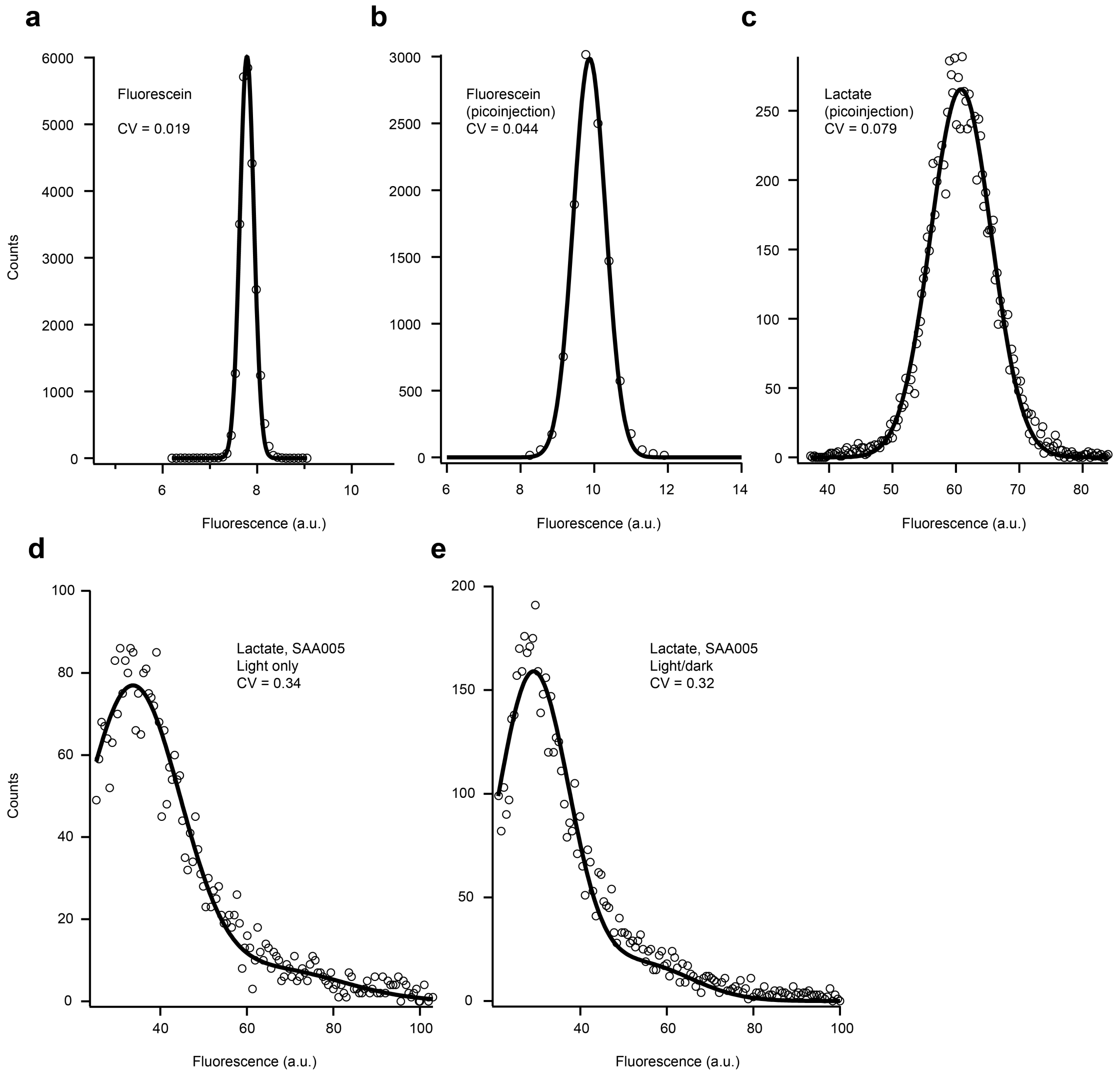

Fig. S2 Variance in data output at different steps of the assay. **(a)** Fluorescein detected after droplet generation (n=25,697 analyzed droplets), **(b)** fluorescein detected after picoinjection of the L-lactate assay (n=10,722), **(c)** the substrate of the L-lactate assay detected after picoinjection (n=10,722), and **(d)** the substrate of the L-lactate assay detected after picoinjection into cell-containing droplets (n=3,277, excluding the empty background-droplets). **(e)** Same experiment as in *d*, except that cells were subject to a 12-hour dark period prior to lactate production (n=5,706). The coefficient of variation (CV) obtained from regression analysis is shown in each panel.

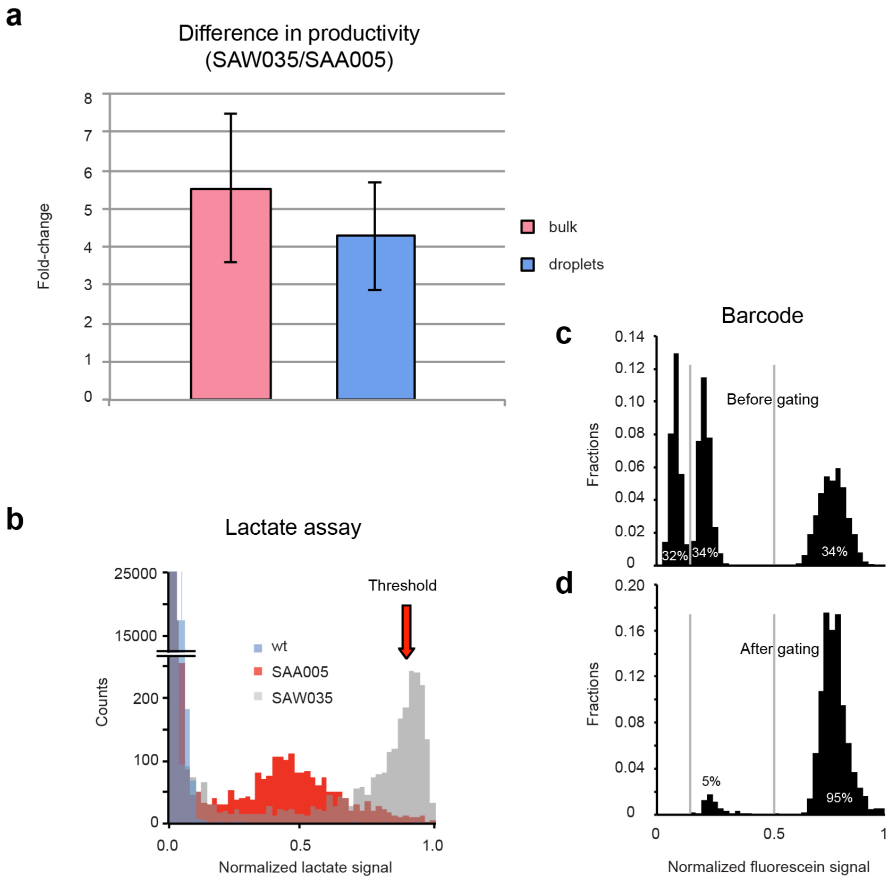

Fig. S3 Barcoding of the assay and differences between strains. **(a)** Productivity difference between SAW035 and SAA005 measured in microtiter plate (pink) and droplets (blue); mean ± s.e.m. (n=3 biological replicates). Fold-change is calculated as $(x_{SAW035}\text{-background}_{WT})/(x_{SAA005}\text{-background}_{wt})$. **(b)** Lactate detected in an equal mix of wild type, SAA005 and SAW035 strains (n={28,648; 31,029; 30,324} is total number of droplets in respective strain; y-axis is broken). The signal is normalized between 0 and 1 on the x-axis. Wild type signal overlaps with empty droplets. **(c)** Fluorescein signal in the same droplets as in *b*, showing distinct populations at concentrations 0, 2 and 10 µM. **(d)** When gating for the top fraction at the threshold indicated by the red arrow in *b*, and measuring the signal of the barcode as in *c*, most of the remaining droplets belong to the top-producing strain SAW035.

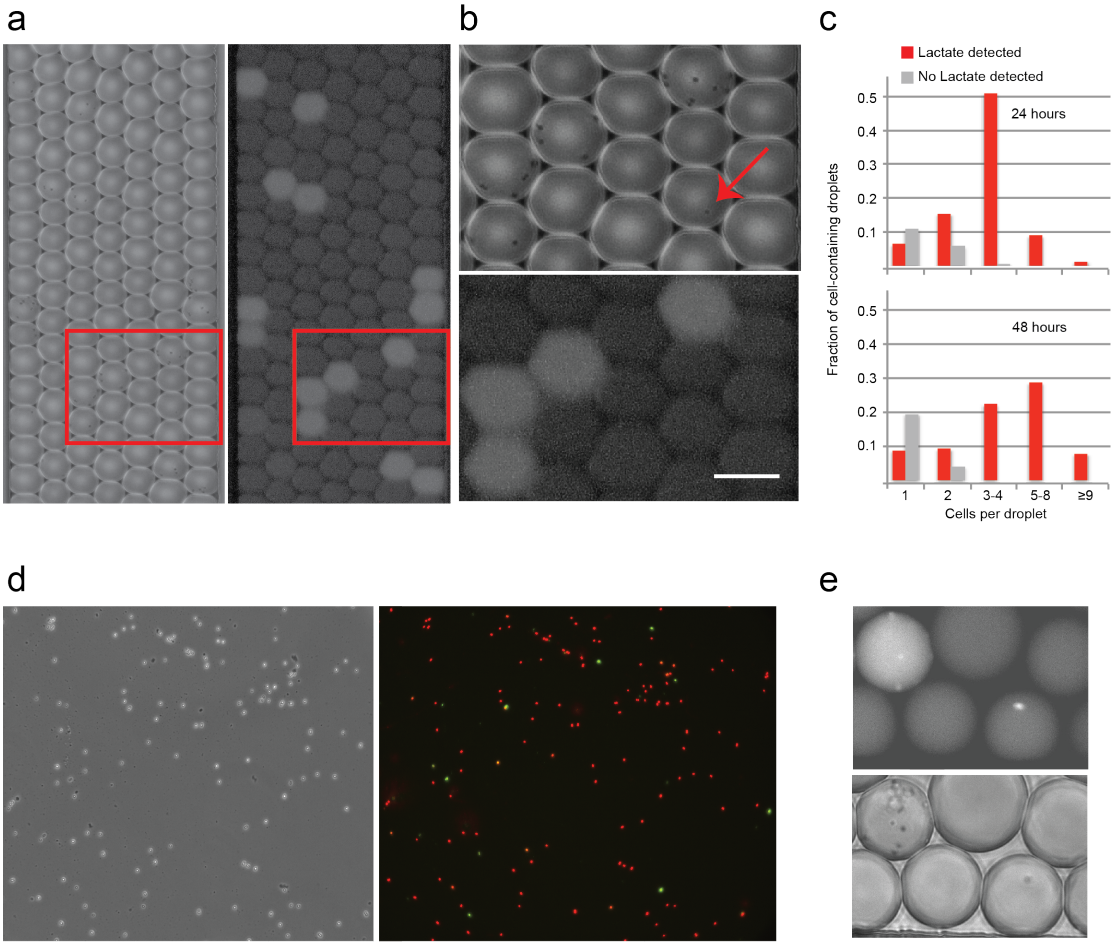

Fig. S4 Detection of cells and extracellular lactate. **(a)** Images show droplets in a microfluidic channel at 10× optical magnification, (*left*) phase contrast, and (*right*) fluorescence channel. **(b)** Magnification of marked regions in *a*, the arrow indicates a droplet with a single cell and no observed fluorescence. Scale bar = 30 µm. **(c)** Number of cells per droplet, in droplets with detected fluorescence (red), and no detected fluorescence but at least one cell observed (grey), measured after 24 (*top*) and 48 hours (*bottom*) incubation for lactate production. N ≥ 1,000 analyzed droplets per time point and of these 15% contain cells. **(d)** Viability assay where dead cells are stained green by DiBAC$_4$(3)/oxonol and live cells are autofluorescent in red due to phycocyanin and chlorophyll. **(e)** Autofluorescence from *Synechocystis* in droplets. A single fluorescent bacterium is seen in a droplet where no lactate is detected, while in a droplet with several bacteria the autofluorescence is barely seen over the signal from the L-lactate assay.

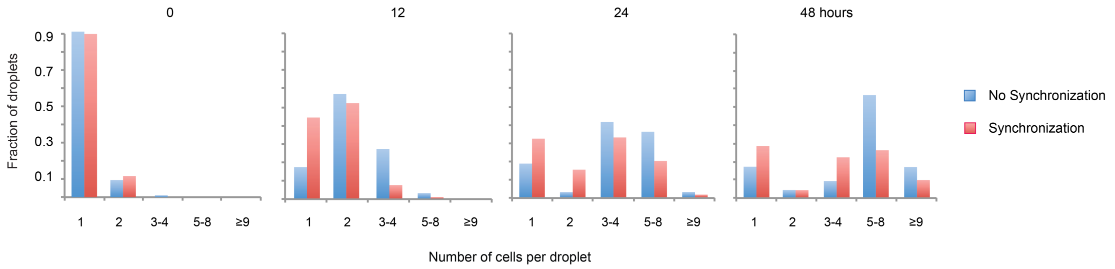

Fig. S5 Cell division of strain SAA005 in droplets with (red) or without (blue) 12 hours pre-incubation in darkness. Distribution of observed number of cells per droplet over 12-48 hours incubation (data is the average of two biological replicates with in total $n_{0,12,24,48,\text{non-sync}}$={108, 219, 400, 335} and $n_{0,12,24,48,\text{sync}}$={105, 200, 325, 376} droplets).

Supplementary Tables

Table S1 Comparison of optical density and cell concentration

Strain	OD	Cells/ml (at OD=1)
SAA005	1.32 ± 0.02	$1.151 \pm 0.040 \times 10^8$
SAW035	0.51 ± 0.08	$1.004 \pm 0.081 \times 10^8$
Wild Type	1.46 ± 0.10	$1.161 \pm 0.173 \times 10^8$

Optical density at 730 nm (OD_{730}) and cell count normalized to OD=1 for SAA005, SAW035 and wild type. Mean ± s.d., (n=3, technical replicates).

Supplementary Data and Notes

1. Growth and viability in droplets

When the encapsulated cells are incubated for sufficient time such that apparently all lactate containing droplets saturates the assay (for example seen for SAW035 in Fig. 1c, and SAA005 in Fig. 2b), we can calculate the fraction of droplets that contain lactate. Since also the number of cells per droplet can be quantified by the time of droplet encapsulation, the numbers can be compared and we get an estimate of the fraction of cells that actually produce lactate to levels above the background. For SAW035 this analysis gives on average 13.5% droplets with detected lactate and 27.3±1.9% (mean ± s.e.m. n=3) of cells that appears not to produce lactate. For SAA005 the fraction of non-producers is significantly lower at 15.9±2.8% (n=5), and also when the cells are subject to a 12-hour dark period at 14.8±4.9% (n=3).

In the same way, by looking at the droplet growth data (Fig. 2a) over a period of 48 hours it is seen that a fraction of the cells divide very slowly or not at all. These fractions are in the range between 10 and 30% in different experiments. This raises the question if non-dividing cells also do not produce lactate, or if there is no such correlation. A simple explanation would be if the corresponding fractions of cells were dead by time of encapsulation in droplets.

Using oxonol-based live/dead staining and fluorescence microscopy (Fig. S4d) we show that the fraction of cells that are apparently non-viable at time of droplet encapsulation is 4.9% (n=350), 6.4% (n=530) and 7.7% (n=773) for SAW035, SAA005 and SAA005 incubated in darkness, respectively. This is further confirmed by the observation that non-stained cells show autofluorescence from the photosynthetic machinery [30] while stained cells have lost the autofluorescence. The results indicate that the fraction of cells that neither divide nor produce detectable lactate include dead cells. However since this fraction of non-viable cells is smaller than the fraction of non-dividers and non-producers, additionally metabolically stalled cells must be present in the population.

We investigated this by fluorescence microscopy, where droplets following addition of the L-lactate assay were re-injected into a microfluidic chip for imaging in one plane (Fig. S4a-c). As seen in Figure S4c there is a strong relationship between cells dividing slowly and no lactate being detected above the background in the droplets. Based on [30] and our observations in Figure S4d we assume that autofluorescence is a phenotype for live cells. The droplets imaged in Figure S4c, containing cells but no lactate, were therefore analyzed for autofluorescent cells (Fig. S4e), and after 24 and 48 hours incubation the fraction of these droplets with autofluorescent (viable) cells were 0.73 and 0.6 respectively. Comparing the number of non-viable cells with the total number of droplets with cells (including dividing and lactate producing) we find that the fractions of dead cells are 4.7% (24 hr) and 8.3% (48 hr). This is similar to the fraction at encapsulation and implies that cells stay viable in droplets.

2. Lactate production in droplets and batch culture

Lactate productivity was evaluated in 96-well microtiter plate by determining the concentration of lactate in the cell suspension after 12-16 hours of incubation. Subtracting the background signal for the wild type reference strain (where the major contribution from the signal comes from assay reagents) we determine a difference in productivity of 5.5±1.9-fold for SAW035 over SAA005. Similarly, the difference between strains in the droplets was quantified by estimating the fluorescence value of the center of the respective peak in the histogram (after gating with the barcode and subtracting the background signal). Here, SAW035 shows an accumulated productivity 4.3±1.4-fold higher than that of SAA005 after 5-6 hours of incubation (Fig. S3a).

The reason why longer incubation times are used in the microtiter measurements is that the cell concentration is 3-4 times lower. This is since encapsulating 1 cell per 10 pL droplet increases the concentrations to approximately OD_{730}=1 as seen in Table S1.

3. Variability in droplet detection

We estimated the coefficient of variation (CV) (Eq. S1)

$$\mathrm{CV} = \frac{\sqrt{v}}{u}, \mathrm{Eq.\,S1}$$

for the datasets of fluorescein and lactate using a single Gaussian function (Eq. S2), where u and v are the fitted mean and variance respectively and a an arbitrary fitted constant.

$$a\left(e^{-(x-u)^2/(2v)}\right), \mathrm{Eq.\,S2}$$

The variance in the droplet microfluidic assay increases with each additional step as illustrated in Figure S4. The distribution in the fluorescein signal following droplet generation depends on variations in droplet size and the fluorescence detection. When picoinjection is performed, the droplet volume doubles and the fluorescein concentration is halved. The increased variance in detected fluorescein signal is due to uneven injection, resulting in differences in droplet size and non-uniform dilution of the fluorophore. The uneven injection means that different amounts of the L-lactate assay mixture are added to different droplets. This together with the assay variability explains why the variance in measured lactate is higher compared to the fluorescein barcode in the same droplets. These three observations (Fig. S2a-c) together describe the main technical variance in our assay.

The additional variance observed for lactate produced by the cells can thus be attributed to biological variance. Since droplets are generated over a time of 20 minutes there will be a difference in incubation time between cells. However, after six hours of incubation this means a difference of only 5%, and this effect is further reduced since all droplets are exposed to the light in the photo-incubator at the same time point. Instead the main explanation in cell-to-cell variations is the state of the cell cycle at the time of droplet encapsulation, which means that different cells have different lactate productivity. There will also be a higher signal in droplets where cells are co-encapsulated.

When cells are encapsulated to produce lactate we anticipate a mixed population in the droplet data. At generation, the number of cells encapsulated per droplet follows a Poisson distribution as long as the cell suspension is evenly mixed [31]. For example, if the average number of cells per droplet is around 0.1, the fraction of droplets with one cell is approximately 10% and the fraction with two cells 0.5%. 90% of the droplets will not contain a cell. The datasets of lactate from cells were therefor fitted to a sum of Gaussian functions (Eq. S3), where the peak corresponding to empty droplets have been excluded from the analysis.

$$a\sum_{1}^{n}\frac{e^{-\lambda}\lambda^{n}}{n!}\frac{1}{\sqrt{n}}e^{-\frac{(x-nu-b)^2}{2nv}}, \mathrm{Eq.\,S3}$$

Here a, u and v are as in Eq. S2, b is a fitted offset, λ is the average number of cells per droplet as quantified by microscopy data, and n=1, 2, ... is the number of cells in one droplet. We here assume that two cells on average produce twice the amount of lactate as one cell. The contribution from $n \geq 3$ is negligible (on average the datasets contain

0.15% droplets with three cells at generation), and data is fitted for the sum from n=1 to 2. Figure S2d, e shows examples of fitted data for SAA005 with and without a 12-hr dark period. The time-points for this analysis were chosen such that the signal was not yet saturated, and at the same time high enough to separate the background from the droplets containing lactate-producing cells. The analysis shows that there is no significant effect from the dark period on the peak widths. The coefficient of variation is 0.35±0.022 (mean ± s.e.m. n=3) for continuous light and 0.31±0.016 (n=3) with the dark period. The datasets were also fitted with the offset fixed at b=0 and this gives 0.33±0.019 and 0.30±0.055 respectively.

4. Quantifying sorting efficiency

As shown in Table S1, optical density (OD) scales differently with cell concentration for the strains, with a difference of about 15 % between SAW035 and SAA005. This is probably related to the difference in growth rate between strains [19]. For this reason OD from spectrophotometer measurements was not used as reference when calculating the enrichment in sorting. Cells were mixed 10:1 based on OD, washed and encapsulated. In addition SAA005 and SAW035 were separately encapsulated at the same OD and imaged directly in the microfluidic chip. This allowed us to perform cell counting specifically for each sorting experiment. Cell counting typically showed a difference of 20-30% between strains in terms of number of observed cells per droplet, close to agreement with the batch culture experiment. These numbers were accounted for when quantifying enrichment in sorting. Enrichment was calculated as this corrected ratio before sorting (for example 12.6:1 in Fig. 2d) divided by the ratio obtained from colony count after sorting (1:2.6 in Fig. 2d). The theoretically maximal enrichment for non-overlapping populations can be estimated as in [17] (Eq. S4):

$$n_{max} = \frac{1}{1 - e^{-\lambda\epsilon_0/(1+\varepsilon_0)}} \text{ , Eq. S4}$$

where λ is the average number of cells per droplet and ε_0 is the ratio between high and low producer before sorting. For the dataset in Figure 2d these numbers are 0.177 and 1/12.6 and the resulting n_{max}=77.

Following sorting three colonies each from selection plates specific for SAA005 and SAW035 respectively were picked, cultured and assayed in microtiter plate for lactate production. It showed 3.2±1.8-fold (mean ± s.e.m.) higher concentration for SAW035 compared to SAA005, which is comparable to the difference measured before sorting (Fig. S3a).

5. Supplementary Methods

Fluorescence microscopy

Fluorescence and phase contrast microscopy was performed using an inverted Nikon Eclipse Ti-E with objective lenses CFI Plan Fluor DL-10X/NA=0.30 or CFI Super Plan Fluor ELWD ADM 40X/NA=0.60, Lumencor SOLA II Solid state White light engine, Andor Zyla sCMOS 5.5 camera and Semrock filter sets "GFP" (Ex 472/30, DM 495, Em 520/35) or "TxRed" (Ex 562/40, DM593, Em 624/40).

Live/dead staining and fluorescence microscopy

Cells were collected from liquid culture and washed twice as described in *Methods* in the main text. The cell pellet corresponding to 0.5 mL culture was re-suspended in 30 µL (1µM) $DiBAC_4(3)$/oxonol (Molecular Probes Life Technologies) which stains non-viable cells as it penetrates damaged cell membranes and binds intracellular proteins [32]. Following 30 minutes incubation a 1 µL aliquot was put between a microscope slide and

a cover slip and stepwise imaged at 40× optical magnification using phase contrast (observe all cells) and fluorescence with "GFP" (stained, dead) and "TxRed" (autofluorescent, live) respectively. The result was quantified as fraction of green and fraction of red cells.

Fluorescence microscopy for lactate production

Cells of strain SAA005 were encapsulated in droplets, incubated for 24 and 48 hours and the L-lactate assay was applied. Following picoinjection droplets were collected, re-injected in another microfluidic chip and analyzed using phase contrast and fluorescence microscopy with the "TxRed" filter set.

Optical density normalization

Cultures were started by picking colonies from fresh plates of wild type, SAA005 and SAW035 and inoculating approximately the same amount of each strain in separate shake flasks in liquid BG-11 with appropriate antibiotics (see *Methods* in the main text). Cells were cultured for several days – in a shaking incubator at 120 rpm and 30°C with a light intensity of 20-25 μE/m^2/s (cool white fluorescent light) – until reaching OD_{730} ~ 1.0 (late exponential growth phase). Cultures were analyzed by cell counting, using a Bürker-Türk hemocytometer and a light microscope with 40× magnification, and measuring optical density.

Microtiter plate measurements

Aliquots of the same samples as encapsulated in droplets were incubated in transparent 2 mL cuvettes in parallel with the droplet emulsions in syringes. 25 μL cell suspension was mixed with 25 μL L-lactate assay (see *Methods* in the main text) in 96-well plates (Greiner) and time-lapse experiments were run using excitation around 490 nm and emission around 590 nm (SpectraMax M5, Molecular Devices). The enzymatic reaction was complete in less than 20 minutes and the plateau level was used as the readout value. Wild type cells were treated, incubated and assayed in the same way to obtain a reference base line and calculate relative differences.

6. Supplementary references only

30. Schulze K, López DA, Tillich UM, Frohme M. A simple viability analysis for unicellular cyanobacteria using a new autofluorescence assay, automated microscopy, and ImageJ. BMC Biotechnol. 2011 Nov 30;11:118

31. Huebner A, Olguin LF, Bratton D, Whyte G, Huck WT, de Mello AJ, Edel JB, Abell C, Hollfelder F. Development of quantitative cell-based enzyme assays in microdroplets. Anal Chem. 2008 May 15;80(10):3890-6

32. Kaczmarzyk D, Anfelt J, Särnegrim A, Hudson EP. Overexpression of sigma factor SigB improves temperature and butanol tolerance of Synechocystis sp. PCC6803. J Biotechnol. 2014 Jul 20;182-183:54-60
